# Association of the Total Cholesterol Content of Erythrocyte Membranes with the Severity of Disease in Stable Coronary Artery Disease

**DOI:** 10.1155/2014/821686

**Published:** 2014-10-20

**Authors:** Gholamreza Namazi, Morteza Pourfarzam, Sabieh Jamshidi Rad, Ahmad Movahedian Attar, Nizal Sarrafzadegan, Masoumeh Sadeghi, Parastoo Asa

**Affiliations:** ^1^Department of Clinical Biochemistry, School of Pharmacy and Pharmaceutical Sciences, Isfahan University of Medical Sciences, Hezar-Jerib Street, Isfahan 8174673461, Iran; ^2^Isfahan Cardiovascular Research Centre, Cardiovascular Research Institute, Isfahan University of Medical Sciences, Isfahan 8187698191, Iran

## Abstract

Increasing evidence suggests that erythrocytes may participate in atherogenesis. We sought to investigate whether the total cholesterol content of erythrocyte membranes (CEM) is significantly different in patients with stable coronary artery disease (CAD) compared to patients with nonsignificant coronary stenosis and determine the correlation between CEM and the severity of coronary stenosis. *Methods*. The population included 144 patients, undergoing clinically indicated coronary angiography. The severity of coronary stenosis was scored after coronary angiography and patients were divided into two groups; the *S*-stenosis group (CAD patients, *n* = 82) had a significant stenosis indicated by coronary angiography and the second group, *N*-stenosis (*n* = 62), had nonsignificant coronary stenosis. Lipid parameters were determined by routine laboratory methods. CEM was measured using an enzymatic assay, and protein content was assessed by the modified Lowry method. *Results.* The mean of CEM levels was higher (*P* < 0.001) in stable CAD patients (137.2 *µ*g/mg of membrane protein) compared with *N*-stenosis patients (110.0 *µ*g/mg of membrane protein). The coronary artery scores were correlated positively with CEM levels (*r* = 0.296, *P* < 0.001). *Conclusion*. CEM levels are positively associated with the severity of CAD, meaning that CEM might contribute to the development of CAD.

## 1. Introduction

Coronary artery disease (CAD) is closely associated with advanced atherosclerosis, which reflects several deteriorative phenomena that gradually result in narrowing of coronary arteries, terminating in thrombosis and myocardial infarction. CAD is one of the major causes of mortality and morbidity in both developed and developing countries and is believed to have a multifactorial etiology, composed of numerous biological, environmental, behavioral, and sociocultural factors [1–3]. In addition to traditional risk factors, erythrocyte membrane has been regarded as one of the most important contributors to the initiation and progression of atherosclerosis [4–11].

Although apoptotic macrophages are an important source of cholesterol within atherosclerotic plaques, it is unlikely that all of the cholesterol contained in plaques derives from foam cells alone. Most of the cholesterol in foam cell is esterified [12], whereas the atherosclerotic lipid core has a remarkably high content of free cholesterol [13]. The most compelling evidence was reported by Arbustini et al. [14], who identified an erythrocyte membrane protein, glycophorin A, in pulmonary plaques from patients with thromboembolic disease. These findings were repeated in coronary plaques from patients with sudden cardiac death, proposing that intraplaque haemorrhage and erythrocytes lysis lead to the accumulation of cholesterol and expansion of the necrotic core [15]. Therefore, it is clear that the amount of erythrocytes incorporated into the plaque, their total lipid composition, and total cholesterol content of erythrocyte membranes (CEM) could influence plaque core size and composition. The CEM has previously been reported as a potential marker of clinical instability in the setting of coronary artery disease (CAD) [16]. However, CEM levels were not associated with the angiographic extent of coronary atherosclerosis [16]. This may seem controversial because the suggested contribution of erythrocyte membrane lipids to atheromatic plaque vulnerability is in part mediated by necrotic core growth [17].

We sought to investigate whether the CEM is significantly different in patients with stable coronary artery disease (CAD) compared to patients with nonsignificant coronary stenosis and determine the correlation between CEM and the severity of coronary stenosis. In addition, we also sought to determine the extent to which this relationship was independent of other potential risk factors associated with CAD.

## 2. Methods

### 2.1. Patient Population

A total of 144 patients who underwent diagnostic coronary angiography in the Department of Cardiology (Noor Hospital, Isfahan, Iran) from August 2012 to December 2012 were included in this case-control study. Patients with a history of diabetes, hematologic diseases, or any other chronic medical illness and those with unstable angina including ST segment elevation myocardial infarction (STEMI), non-ST segment elevation myocardial infarction (non-STEMI), and unstable angina within the period of one month prior to the commencement of study were excluded from the study. The study protocol was approved by the ethics committee of Isfahan University of Medical Sciences and written informed consent was obtained from each participant.

### 2.2. Anthropometric Measurements

Weight was measured, with subjects minimally clothed without shoes, and height was measured in a standing position. Body mass index (BMI) was calculated as weight in kilograms divided by height in meters squared. Waist circumference (WC) was measured at the narrowest level and that of hip at the maximal level over light clothing, using a tape measure. Waist hip ratio (WHR) was calculated as WC divided by hip circumference. To avoid subjective error, all the measurements were taken by the same person. Blood pressure was measured by a qualified physician after a rest for 15 min [18]. Study participants were deemed hypertensive if they had a systolic pressure ≥140 mm Hg and/or diastolic pressure ≥90 mm Hg and/or were already taking any form of antihypertensive medication.

### 2.3. Angiographic Analyses

Two experienced cardiologists, unaware of the patient's clinical history and biochemical results, evaluated all angiograms to assess the severity of the CAD. The severity of coronary stenosis was scored based on coronary angiography data and a numerical score from 0 to 21 was allocated to each patient [19]. Patients were divided into two groups according to their CAD scores; the *S*-stenosis group (CAD patients, *n* = 82) had a significant coronary stenosis (score >  7) and the second group, *N*-stenosis (*n* = 62), had nonsignificant stenosis (score ≤ 7) indicated by coronary angiography.

### 2.4. Collection and Preparation of Blood Samples

Venous blood samples were collected after overnight fasting from median cubital vein of all study subjects in heparinized tubes. In order to prepare packed erythrocyte and isolate plasma, blood was centrifuged at 1500 g for 10 min. The resulting plasma was removed, divided into 0.5 mL aliquots and stored at −80°C for future assays. The remaining RBCs were resuspended and washed twice in Tris-buffered saline (TBS) (20 mM Tris-HCl, pH 7.5 containing 145 mM NaCl). The washed packed cells were collected by centrifugation at 2000 g for 15 min [20] and washed packed erythrocytes were used for erythrocytes membrane isolation.

### 2.5. Biochemical Analysis

Plasma levels of hsCRP were measured with a latex-enhanced immunoturbidimetric assay (Roche Diagnostics, Mannheim, Germany) on a Hitachi 902 Automatic Analyzer (Roche Diagnostics, Indianapolis, IN, USA). Fasting blood glucose, triglyceride, total cholesterol, HDL-C, and LDL-C were determined using standard enzymatic kits (Parsazmun, Tehran, Iran) on the Hitachi 902 Automatic Analyzer.

### 2.6. Isolation of Erythrocyte Ghost Membrane

Erythrocyte ghost membrane was prepared by hypotonic lysis of packed cells. 2.5 mL of the washed packed erythrocytes was mixed with 10 volumes of ice cold 5 mM Tris and 0.1 mM Na_2_EDTA (pH 7.6). The mixture was gently swirled and allowed to stand for 15 min at 4°C. The hemolysate was then centrifuged at 20000 g for 20 min at 4°C and supernatant was discarded. The pellet was washed three times with (20 mL) of 17 mM NaCl and 5 mM Tris-HCl (pH 7.6) and then three times with (20 mL) of 10 mM Tris-HCl (pH = 7.5) and after centrifugation at 20000 g the pellet was resuspended in 10 mM Tris-HCl (pH = 7.5) [21]. Multiple aliquots (0.2 mL) of this hemoglobin-free membrane suspension were immediately frozen in liquid nitrogen and stored at −70°C until assay of membrane protein and CEM. Protein concentration in erythrocyte membrane was measured by the method of Lowry as modified by Markwell et al. [22].

### 2.7. Erythrocyte Membrane Lipid Extraction

Lipids were extracted from the erythrocyte membrane samples according to the Folch method [23]. Briefly, 1 mL of chloroform-methanol (2 : 1 v/v) was added to 100 *μ*L of erythrocyte membrane suspension in a leak proof test tube. Tubes were vortexed vigorously for 1 min. Following this, 500 *μ*L of 0.9% NaCl solution was added and tubes were mixed again. Tubes were then centrifuged at 1000 rpm for 3 min to separate the phases. The lower chloroform phase (containing lipids) was collected and transferred a 1.5 mL microfuge tube and dried carefully under nitrogen gas. The dry residue was dissolved in 100 *μ*L isopropanol, and its total cholesterol content was determined using a commercial enzymatic assay (Pars-Azmoon, Tehran, Iran). Results are expressed as micrograms of total membrane cholesterol per milligram of membrane protein.

### 2.8. Statistics

All the statistical analyses were performed using the SPSS for Windows, version 16 software package (SPSS Inc., Chicago, IL, USA). Results for continuous variables are presented as means and SD or as medians and interquartile ranges if the distributions were skewed and as percentages for categorical data. Data were tested for normal distribution with the Kolmogorov-Smirnov test. The independent *t*-test or the Mann-Whitney *U* test were used to evaluate differences in continuous variables between the two groups. Comparisons between categorical variables were performed with the Chi-square test. Correlations between variables were studied by Pearson correlation. Differences were considered significant when *P* value was <0.05. We used multivariable linear regression models to determine the relationship, expressed as *β*-coefficients, between CEM levels and CAD score. Multivariate adjustment was made for age, sex, hypertension, current smoking, hematological indices, hsCRP, triglyceride, LDL-cholesterol, HDL-cholesterol, statin use, aspirin use, BMI, and WHR.

## 3. Results

**Figure 1:**
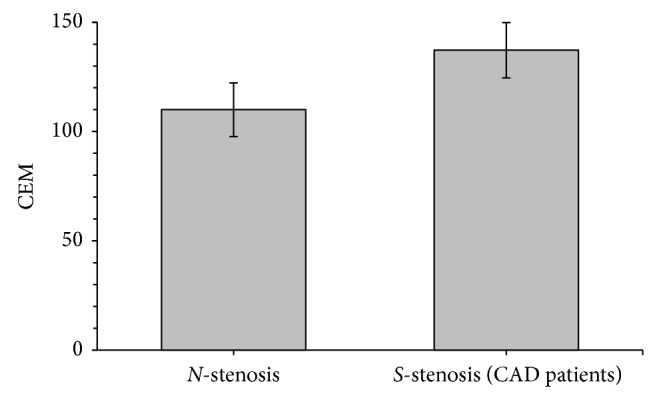
CEM level in the *S*-stenosis (CAD patients) and *N*-stenosis patients. Error bars represent the 95% confidence interval. *N*-stenosis refers to the patients with nonsignificant coronary stenosis (score ≤ 7); *S*-stenosis refers to the patients with significant coronary stenosis (score > 7).

**Figure 2:**
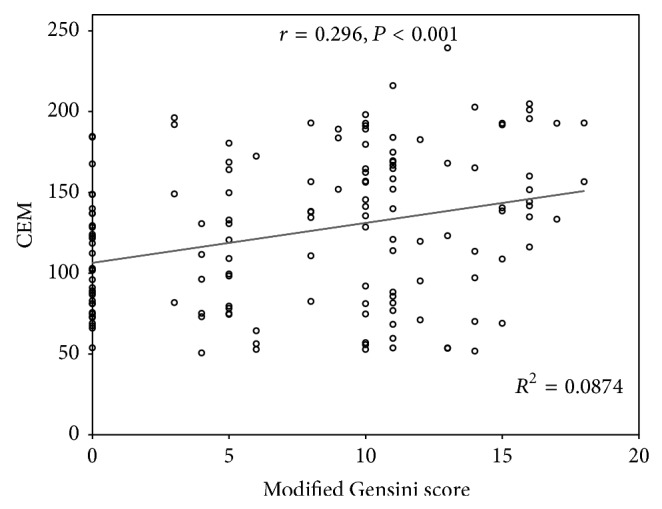
Correlation of coronary stenosis scores with CEM in the whole study subjects.

Results of the anthropometric, biochemical, and clinical variables in the stable CAD patients (*S*-stenosis) and *N*-stenosis patients are summarized in Table 1. No significant differences were observed in age, BMI, WHR, hypertension, and smoking between the two groups. The medications taken by both groups at the study entry were similar with the exception of *β*-blockers, statins, nitrates, aspirin, and clopidogrel. As expected, the number of subjects taking these medications was significantly higher in the stable CAD patients (*S*-stenosis). Total cholesterol and LDL cholesterol levels were significantly lower in patients with CAD in comparison with *N*-stenosis patients. In our study, 79% of patients in the CAD group were using statins, which was higher than *N*-stenosis group (39%), and, obviously, this might explain why the total cholesterol and LDL-C levels in the CAD group were lower than *N*-stenosis group. Nonetheless, other biochemical results, including fasting blood glucose, triglyceride, and HDL cholesterol were not significantly different from those in the *N*-stenosis patients. The CEM was significantly higher (*P* < 0.001) in CAD (137.2 *μ*g/mg of membrane protein) patient group compared to *N*-stenosis group (110.0 *μ*g/mg of membrane protein) (Figure 1). In the whole study subjects (*n* = 144), CEM levels showed a positive correlation (*r* = 0.296, *P* < 0.001) with CAD scores (Figure 2). In multivariable-adjusted linear regression models, after sequential adjustment for demographics, cardiovascular risk factors, and medication use, BMI, and WHR, CAD score remained directly associated with CEM levels (Table 2). CEM did not correlate with plasma concentration of hsCRP (*r* = −0.091, *P* = 0.280), total cholesterol (*r* = −0.137, *P* = 0.103), low-density lipoprotein cholesterol (*r* = −0.125, *P* = 0.136), or triglycerides (*r* = 0.64, *P* = 448). However, a significant negative correlation was found between CEM and plasma high-density lipoprotein cholesterol levels (*r* = −0.171, *P* = 0.040).

As the possibility that statin use could influence the association observed between CEM and plasma total cholesterol could not be excluded, we repeated the linear correlation analysis in a subgroup of patients who were not receiving statin treatment (*n* = 55). We found that there was no linear association between CEM and plasma total cholesterol (*r* = −0.169; *P* = 0.217).

## 4. Discussion

In the present study, we observed that the CEM levels were significantly increased among CAD patients compared with *N*-stenosis patients. The mechanisms responsible for increased CEM in patients with stable CAD require investigation. Increased cholesterol uptake by the erythrocyte membrane, reduced cholesterol efflux, or both may be responsible. Furthermore, we observed that CEM was directly associated with the severity of the disease as judged by the modified Gensini scores. This finding is in agreement with previous studies showing positive correlations between CEM levels and the number of diseased coronary arteries [7, 24]. These results are not in agreement with those of Tziakas et al. [16] and Yu et al. [25], who concluded that CEM could be a marker of ACS but not of atherosclerosis.

Tziakas et al. [16] reported that CEM levels were not associated with the extent and severity of coronary artery disease. However, a significant relationship was noted between CEM levels and angiographically complex coronary lesions [16]. The cause for the inconsistency is uncertain, but it could relate to differences in methods of evaluation of the coronary stenosis and the measure for diagnosis of the CAD. Thus, the complicated relationship between CEM and the severity of CAD merits large-scale clinical trials.

In this study, we analyzed the correlation between CEM and lipid elements in the plasma of patients with CAD. It was found that CEM levels were negatively correlated with plasma HDL levels (*r* = −0.179; *P* = 0.040), which suggested that the decrease in HDL levels may be involved in increase in CEM levels. In our study, however, circulating cholesterol levels did not correlate with CEM content, probably because CEM content represents the equilibrium achieved between erythrocyte influx and efflux, and intracellular cholesterol transport [26]. Several biochemical and enzymatic steps are involved in the handling of cholesterol by the erythrocyte, which are regulated by complex feedback mechanisms and genetic factors that regulate protein expression and enzymatic activity [27].

Therefore, it appears that the increased cholesterol levels in erythrocyte membrane do not simply reflect an equilibrium between the plasma and membrane cholesterol concentrations at any particular time. Instead, CEM levels most likely reflect lipid profile over a long period of time as glucosylated hemoglobin (HbA1c) levels reflect glucose control over a long period of time.

The findings of the present study should be interpreted in light of certain limitations. First, our research represents a small observational study; however, it was powered enough to detect differences in CEM levels among study groups. Even with the relatively small sample size, the present study is hypothesis-generating. Larger studies are required to investigate further the predictive value of CEM. Second, underlying pathogenetic mechanisms are still speculative. Third, we measured total and not free cholesterol content of erythrocyte membranes. Finally, the association of CEM with the severity of disease in stable coronary artery disease is not supported by plaque imaging data such as intravascular ultrasound (IVUS) data. Imaging studies are required to establish further the pathogenetic implication of CEM in stable CAD patients.

## 5. Conclusion

Taken together, the findings of the present study, notwithstanding the acknowledged limitations, extend the current understanding of the widely discussed atherogenic impact of erythrocytes, by demonstrating a significant correlation between CEM levels and severity of CAD in patients with angiographically documented CAD. Furthermore, these observations may suggest that the measurement of plasma cholesterol levels alone may not be accurate to study lipid profile in CAD patients, since they may be affected by short term changes in the dietary or environmental factors. On the other hand, the measurement of CEM may be helpful as additional risk factors in ratification and management of CAD patients. However, further studies are required to establish the clinical importance and pathogenic significance of CEM measurement.

## Figures and Tables

**Table 1:** Anthropometric, biochemical, and clinical data in CAD patient (*S*-stenosis) and *N*-stenosis group.

	*N*-stenosis patients (*N* = 62)	CAD patients (*S-*stenosis) (*N* = 82)	*P* value
Demographic data			
Age (years)	55.1 ± 6.2	56.1 ± 6.8	0.363
Men/women (*n*)	42/20	61/21	0.381
Body mass index (kg/m^2^)	28.1 ± 3.8	28.0 ± 4.6	0.868
Waist hip ratio	0.91 ± 0.05	0.91 ± 0.06	0.868
Systolic pressure (mm Hg)	129 ± 19	133 ± 23	0.244
Diastolic pressure (mm Hg)	87 ± 12	87 ± 15	0.934
Full blood count analysis			
Erythrocyte count (×10^6^/*μ*L)	5.12 ± 0.44	5.15 ± 0.43	0.688
Haematocrit (%)	44.15 ± 3.86	44.35 ± 3.81	0.760
Hemoglobin (g/dL)	15.21 ± 1.48	15.18 ± 1.47	0.916
MCV	85.40 ± 5.19	84.85 ± 4.65	0.498
RDW	13.4 (13–13.9)	13.5 (13–13.9)	0.526
White blood cell count (×10^3^/*μ*L)	6.57 ± 1.62	6.98 ± 2.02	0.199
Risk factors			
Hypertension, *n* (%)	41 (66%)	58 (71%)	0.555
Smoking, *n* (%)	13 (21%)	17 (21%)	0.972
History of ACS, *n* (%)	—	27 (33%)	—
History of coronary intervention			
CABG, *n* (%)	—	6 (7.3%)	—
PCI, *n* (%)	—	12 (14.6%)	—
Medications			
Aspirin, *n* (%)	37 (59.7)	82 (87.8)	<0.001^†^
Nitrates, *n* (%)	14 (23%)	42 (51%)	<0.001^†^
Beta-blockers, *n* (%)	25 (41%)	63 (77%)	<0.001^†^
Clopidogrel, *n* (%)	20 (32%)	47 (57%)	0.003^†^
Statins, *n* (%)	24 (39%)	65 (79%)	<0.001^†^
Biochemistry			
Fasting blood glucose (mmol/L)	5.05 ± 0.72	5 ± 0.72	0.681
Triglycerides (mmol/L)	1.57 ± 0.56	1.57 ± 0.52	0.969
Total cholesterol (mmol/L)	4.97 ± 0.95	4.46 ± 0.99	0.002∗
HDL cholesterol (mmol/L)	1.26 ± 0.23	1.18 ± 0.26	0.072
LDL cholesterol (mmol/L)	2.71 ± 0.67	2.39 ± 0.69	0.005∗
hsCRP (mg/L)	1.4 (0.8–3.3)	1.1 (0.6–3.5)	0.322

*N*-stenosis refers to the patients with nonsignificant coronary stenosis (score ≤ 7); *S*-stenosis refers to the patients with significant coronary stenosis (score > 7). Values are expressed as mean ± SD or median and interquartile range for continuous variables, and as number of patients and % for categorical variables. ∗For independent *t*-test; ^†^for chi-square test. ACE: Angiotensin Converting Enzyme; Ag: Angiotensin; CABG: Coronary Artery Bypass Graft; CAD: Coronary Artery Disease; HDL: High Density Lipoprotein; hsCRP: high-sensitivity C-Reactive Protein; LDL: Low Density Lipoprotein; MCV: Mean Corpuscular Volume; PCI: Percutaneous Coronary Intervention; RDW: Red blood cell Distribution Width.

**Table 2:** Multiple linear regression models showing the relationship, expressed as *β*-coefficients, between CAD score and CEM, after sequential adjustment for potential confounding variables.

	Model 1∗			Model 2^+^			Model 3^++^		
	*β*-coefficient	*R* square	*P* value	*β*-coefficient	*R* square	*P* value	*β*-coefficient	*R* square	*P* value
CEM	0.284	0.123	<0.001	0.271	0.455	<0.001	0.276	0.459	<0.001

^*^Adjusted for Age and Gender.

^+^Adjusted for all variables in Model 1 + hypertension, smoking, hematological indices, hsCRP, cholesterol, triglyceride, LDL-cholesterol, HDL-cholesterol, and medication use.

^++^Adjusted for all variables in Model 2 + BMI and WHR.
